# Expression of the retinoic acid catabolic enzyme CYP26B1 in the human brain to maintain signaling homeostasis

**DOI:** 10.1007/s00429-015-1102-z

**Published:** 2015-09-15

**Authors:** Patrick N. Stoney, Yara D. Fragoso, Reem Bu Saeed, Anna Ashton, Timothy Goodman, Claire Simons, Mohamed S. Gomaa, Angelo Sementilli, Leonardo Sementilli, Alexander W. Ross, Peter J. Morgan, Peter J. McCaffery

**Affiliations:** Institute of Medical Sciences, University of Aberdeen, Aberdeen, AB25 2ZD UK; Department of Neurology, Universidade Metropolitana de Santos, Santos, SP Brazil; School of Biological Sciences, University of East Anglia, Norwich, NR4 7TJ UK; Medicinal Chemistry Division, Welsh School of Pharmacy, Cardiff University, King Edward VII Avenue, Cardiff, CF10 3NB UK; Department of Physiopathology, Universidade Metropolitana de Santos, Santos, SP Brazil; Rowett Institute of Nutrition and Health, University of Aberdeen, Greenburn Road, Bucksburn, Aberdeen, AB21 9SB Scotland, UK; Pharmaceutical Chemistry Department, Faculty of Pharmacy, Suez Canal University, Ismailia, Egypt

**Keywords:** Retinoic acid catabolism, CYP26A1, CYP26B1, Gradient, Hippocampus, Suprapyramidal, Infrapyramidal, Dentate gyrus

## Abstract

Retinoic acid (RA) is a potent regulator of gene transcription via its activation of a set of nuclear receptors controlling transcriptional activation. Precise maintenance of where and when RA is generated is essential and achieved by local expression of synthetic and catabolic enzymes. The catabolic enzymes Cyp26a1 and Cyp26b1 have been studied in detail in the embryo, where they limit gradients of RA that form patterns of gene expression, crucial for morphogenesis. This paracrine role of RA has been assumed to occur in most tissues and that the RA synthetic enzymes release RA at a site distant from the catabolic enzymes. In contrast to the embryonic CNS, relatively little is known about RA metabolism in the adult brain. This study investigated the distribution of *Cyp26a1* and *Cyp26b1* transcripts in the rat brain, identifying several novel regions of expression, including the cerebral cortex for both enzymes and striatum for *Cyp26b1*. In vivo use of a new and potent inhibitor of the Cyp26 enzymes, ser 2–7, demonstrated a function for endogenous Cyp26 in the brain and that hippocampal RA levels can be raised by ser 2–7, altering the effect of RA on differential patterning of cell proliferation in the hippocampal region of neurogenesis, the subgranular zone. The expression of *CYP26A1* and *CYP26B1* was also investigated in the adult human brain and colocalization of CYP26A1 and the RA synthetic enzyme RALDH2 indicated a different, autocrine role for RA in human hippocampal neurons. Studies with the SH-SY5Y human neuroblastoma cell line implied that the co-expression of RA synthetic and catabolic enzymes maintains retinoid homeostasis within neurons. This presents a novel view of RA in human neurons as part of an autocrine, intracellular signaling system.

## Introduction

Retinoic acid (RA), synthesized from vitamin A through a series of oxidative steps, is a lipid regulator of transcription and is the mediator of the majority of vitamin A’s functions. In addition to the enzymes that synthesize RA, the binding proteins transporting RA in the cytoplasm and the nuclear retinoic acid receptors (RARs) that transduce the signal, the capacity to switch off the RA signal is vital for the action of the RA signaling pathway. This task is performed by a subset of the cytochrome P450 family: Cyp26a1, Cyp26b1 and Cyp26c1 (McCaffery and Simons 2007; Ross and Zolfaghari 2011). These microsomal enzymes oxidize RA to a range of metabolites including 4-hydroxy-RA, 18-hydroxy-RA, and 4-oxo-RA (Chithalen et al. 2002). The essential need to turn off the RA signal is illustrated by the embryonic or early postnatal lethality of *Cyp26a1* or *Cyp26b1* null mutations. Loss of *Cyp26a1* results in disruption of the posterior end of the embryo and a caudal regression phenotype, as well as disorganization of the normal patterning of the hindbrain (Abu-Abed et al. 2001). Null mutation of *Cyp26b1* leads to defects in limb development, among other abnormalities (Yashiro et al. 2004). *Cyp26b1* is also required to control timing of meiosis in germ cells of the developing mouse (Bowles et al. 2006). In contrast to its counterparts, *Cyp26c1* is expressed to a lesser extent in the embryo and its loss in the mouse has few effects, although mutation in the human results in a range of facial skin defects (Slavotinek et al. 2013).

It is generally thought that the principal role for the Cyp26 enzymes in embryonic development is to limit the diffusion of RA synthesized from vitamin A, creating regions of high and low RA concentration. This controls the patterns of gene expression that guide development (McCaffery and Simons 2007; Ross and Zolfaghari 2011). In the embryo, the enzymes that synthesize RA from vitamin A (retinol) are primarily retinol dehydrogenase 10 (Rdh10), oxidizing retinol to retinaldehyde, which is then further oxidized to RA by retinaldehyde dehydrogenases 1, 2 and 3 (Raldh1, 2, 3; also known as Aldh1a1, 1a2, 1a3) (Napoli 2012). Swindell and coworkers published a general observation that the regions of RA synthesis in the embryo do not overlap with the areas of RA degradation, which then creates the patterns of RA distribution (Swindell et al. 1999). This implies that RA predominantly acts as a paracrine factor in the developing embryo. Is this the case for its later function in the adult? This new study examined such properties in the adult brain and supports previous research indicating that RA can have such a paracrine function in regions such as the rodent hippocampus, where it performs a number of key functions (Aoto et al. 2008; Chen and Napoli 2008). Surprisingly, it was also found that RA can function in an autocrine manner in the adult human brain, with co-expression of the RA synthetic and catabolic enzymes in the same cell controlling intracellular RA concentration and signaling. This reveals an important aspect of the flexibility of RA signaling in the nervous system.

## Methods

### Tissues

The experiments using human tissue were approved by the Ethics Committee of the Universidade Metropolitana de Santos, SP, Brazil, and by the Brazilian Health Research Committee on April 4th 2011, under the number CONEP 16168, documents registered under the number 25000.169694/2010-18. Samples of liver, cerebellum and caudal human hippocampus from six male individuals aged 55 years or less, who did not present any neurological or psychiatric disease, were collected during post mortem procedures. Brains from individuals whose death was related to head trauma, extensive infection, or toxic, anoxic or metabolic injuries were excluded from this study. Samples from the hippocampus, measuring typically 5 mm on each side, were fixed by immersion in 10 % phosphate-buffered formalin within 24 h of death and processed into paraffin wax blocks within the following 24 h. Other samples were collected in RNA*later* RNA Stabilization Reagent (Qiagen) and stored at 4 °C for qPCR or were fresh-frozen on dry ice and stored at −80 °C for analysis by western blot.

All animal procedures were carried out in accordance with UK Home Office regulations on laboratory animal use according to the Animals (Scientific Procedures) Act 1986 and local ethics committee guidelines.

### Detection of Cyp26 expression by polymerase chain reaction (PCR)

Male and female adult rats were euthanized by overdose of pentobarbital and their brains removed and placed on ice. Medial cortex, lateral cortex, hippocampus, striatum, thalamus, hypothalamus, olfactory bulb, cerebellum, choroid plexus, and meninges were dissected and rapidly frozen on dry ice. Total RNA was extracted from the tissues using a Qiagen RNeasy kit. cDNA was synthesized from 500 ng of RNA using a High Capacity RNA-to-cDNA kit (Applied Biosystems) according to the manufacturer’s protocol. Primers used were *Cyp26a1* (NM_130408.2): For, TTC GGG TGG CTC TGA AGA CT, Rev, CCT CTG GAT GAA GCG ATG TAA AT. *Cyp26b1* (NM_181087.2): For, CCA GGA CTG TAT GCC CAT GA, Rev, CCA CTC ACC AAC AAA AAG ACA AG. All PCR primers used in this study were designed using PrimerBLAST (Ye et al. 2012), apart from rat *Cyp26b1* primers, which were designed and supplied by PrimerDesign Ltd. PCR products were visualized by agarose gel electrophoresis and UV transillumination.

### Detection of Cyp26b1 expression by in situ hybridization

The *Cyp26b1* riboprobe template was prepared as previously described (Helfer et al. 2012) from a 925 bp cDNA fragment corresponding to 2507–3431 bp of the mouse *Cyp26b1* cDNA sequence (NM_001177713.1). *In situ* hybridization was carried out as previously described using ^35^S-labeled riboprobe (Ross et al. 2009).

### In vivo inhibition of CYP26 activity

The CYP26 inhibitor ser 2–7 (methyl 3-(1*H*-imidazol-1-yl)-2,2-dimethyl-3-(4-(naphthalen-2-ylamino)phenyl)propanoate; Gomaa et al. 2011) was dissolved at 8 mg/ml in DMSO. Immediately before use, the ser 2–7 solution was mixed with an equal volume of sterile phosphate-buffered saline (PBS) to give a 4 mg/ml solution. For measurement of retinoic acid levels in tissue, female C57BL/6 mice between 8 and 12 weeks of age received a single intraperitoneal (IP) injection of 10 mg/kg ser 2–7. Control animals received an intraperitoneal (IP) injection of an equivalent volume of 1:1 PBS/DMSO. Treated animals were euthanized using pentobarbital 2, 6 or 18 h after ser 2–7/vehicle administration and the hippocampi rapidly dissected on ice and snap frozen on dry ice. Samples were stored at −80 °C before use.

For cell proliferation studies, RARE-*LacZ* mice (Rossant et al. 1991) between 8 and 12 weeks of age received 10 mg/kg ser 2–7 by IP injection once every 24 h for 3 days. Injections were carried out in the morning on each day. On the third day, immediately after the last ser 2–7 treatment, all mice received three additional injections, 2 h apart, of 50 mg/kg bromodeoxyuridine (BrdU) in PBS. 2 h after the final BrdU injection, the mice were perfused transcardially first with PBS, then 4 % paraformaldehyde (PFA) in phosphate buffer. The brain was removed and placed at 4 °C in PFA overnight, then transferred to 30 % sucrose at 4 °C.

### Measurement of RA levels in tissue

A reporter gene assay was used as described elsewhere (Helfer et al. 2012) to measure levels of RA activity in the hippocampus of ser 2–7-injected mice. Sil-15 cells carrying a *LacZ* reporter gene driven by multiple retinoic acid response elements (Wagner et al. 1992) were maintained in DMEM/F-12 containing 10 % fetal calf serum and 0.8 mg/ml G418. Hippocampi from ser 2–7-/vehicle-injected mice were homogenized under low light conditions in an ice-cold 2:1 mix of isopropanol/ethanol containing 1 mg/ml butylated hydroxytoluene to reduce oxidation. Homogenates were centrifuged at 13,000 rpm for 5 min at 4 °C. The supernatant was diluted 1:50 in DMEM/F-12 and added to a 96-well plate containing Sil-15 cells followed by overnight incubation at 37 °C, 5 % CO_2_. The cells were fixed in 1 % glutaraldehyde and β-galactosidase activity was quantified using X-gal and colorimetric analysis. The response of Sil-15 cells to tissue extracts was compared to a standard curve plotted from the response to a series of known RA concentrations from 1 μM to 1 pM.

### Immunohistochemical measurement of cell proliferation using bromodeoxyuridine (BrdU)

Perfused mouse brains were cut into 40 μm coronal sections using a cryostat. Sections were washed in PBS and then incubated in 1 M hydrochloric acid for 30 min at 47 °C, followed by further washes in PBS. Sections were then blocked in PBS containing 10 % goat serum and 0.3 % Triton X-100 for 2 h at room temperature and incubated overnight at 4 °C in anti-BrdU antibody (AbD Serotec) diluted 1:200 in blocking buffer. After washing in PBS containing 0.3 % Triton X-100 (PBST), sections were incubated in fluorescent anti-rat secondary antibody (1:300 in blocking buffer; Jackson ImmunoResearch) for 2 h at room temperature. Following further washes in PBST, sections were mounted onto slides using mounting medium containing bisbenzimide. Images were obtained by fluorescence microscopy and BrdU incorporation was quantified by counting of the number of BrdU-positive cells in every 12th section from each brain (sections were approximately 480 μm apart). The ratio between the total number of BrdU-positive cells in the supra- and infrapyramidal blades was calculated. Only cells within two cell diameters of the subgranular zone (SGZ; Kuhn et al. 1997) were included in cell counts.

### Quantitative PCR

RNA was extracted from samples of human hippocampus, cerebellum and liver and cDNA synthesised as described above. *GAPDH* was used as a reference gene. Primer sequences: *CYP26A1* (NM_000783.3, NM_057157.2): For, CAC CGT ACG GGT GAT GGG CG, Rev, GCT GGC CAG TGG ACC GAC AC; *CYP26B1* (NM_001277742.1, NM_019885.3): For, ACC GGC CAC TGG CTG CTG, Rev, ACG TTG ATG GCC TCG GGG TG. *GAPDH* (NM_002046.5): For, TCT TTT GCG TCG CCA GCC GA, Rev, AGT TAA AAG CAG CCC TGG TGA CCA.

### Western blotting

Human hippocampus was homogenized in 0.01 M phosphate buffer, pH 7.0, containing a broad spectrum protease inhibitor cocktail (Roche) using mechanical homogenization and three freeze–thaw cycles. Homogenates were centrifuged for 10 min at 12,000 rpm at 4 °C. Total protein levels in each sample were quantified using the bicinchoninic acid assay (Pierce) and 50 μg protein was loaded per lane of a 12 % SDS-PAGE mini-gel. After separation, the proteins were transferred onto a Hybond-ECL nitrocellulose membrane (GE Healthcare) using a Mini Trans-Blot Cell (Bio-Rad) and equal loading was confirmed with Ponceau-S (Sigma). Membranes were blocked for 1 h at room temperature in TBS containing 0.05 % Tween-20 (TBST) and 5 % skimmed milk and then probed with mouse anti-CYP26A1 antibody (1:1000 in blocking buffer; Vertebrate Antibodies) at 4 °C overnight. Membranes were washed in TBST and then incubated in HRP-conjugated anti-rabbit secondary antibody (1:3000; Jackson ImmunoResearch) for 1 h at room temperature. The membranes were washed again in TBST and antibody binding visualized by enhanced chemiluminescence (ECL; Millipore) and exposure to X-ray film (Thermo).

### Detection of CYP26 in the human brain by immunohistochemistry

Formalin-fixed samples of human hippocampus were processed into paraffin wax blocks and sectioned at 7 μm. Sections were mounted on TruBond 380 slides (Electron Microscopy Sciences) and dried overnight at 37 °C. Sections were dewaxed in xylene and rehydrated through a series of decreasing ethanol concentrations (100, 95, 80 and 70 %). The slides were washed briefly in PBS, pH 7.4, and then boiled for 10 min in sodium citrate buffer, pH 6.0. After cooling, slides were washed in PBS containing 1 % Tween-20 (Sigma) and 1 % pooled human serum (Bio-Sera), hereafter referred to as PBS(HS). Tissue sections were incubated for 1 h at room temperature in blocking solution consisting of PBS, pH 7.4, containing 0.3 % Tween-20, 5 % goat serum, 5 % bovine serum albumin and 5 % pooled human serum (Makitie et al. 2013). Sections were then incubated with primary antibodies (chicken anti-MAP2, 1:400, Abcam; goat anti-RALDH2, 1:1000, Santa Cruz; mouse anti-CYP26A1, 1:50 Vertebrate Antibodies; goat anti-Iba1, 1:300, Abcam) diluted in blocking solution and incubated overnight at 4 °C. After incubation, the slides were washed in PBS(HS). The tissue was then incubated for 1 h at room temperature in appropriate fluorescent secondary antibodies (Jackson ImmunoResearch) diluted 1:300 in PBS(HS). Slides were washed in PBS(HS) and incubated for 1 min with 10 % Sudan black (Acros Organics) in 70 % isopropanol to reduce auto-fluorescence (Schnell et al. 1999; Neumann and Gabel 2002). The slides were then thoroughly washed in distilled water and mounted with mounting medium containing bisbenzimide (Sigma). The quantitation of number of labeled cells was based on counts of between 60 and 90 cells.

### SH-SY5Y treatment with vitamin A and retinoic acid

SH-SY5Y neuroblastoma cells were maintained in DMEM/F12 containing 10 % fetal calf serum and penicillin/streptomycin and Glutamax (Invitrogen). Cells were treated with either 0.35 μM retinol and 0.3 μM retinyl acetate or 1 μM retinoic acid for 8 or 24 h with serum replaced with B27 supplement (Gibco). Retinoic acid was dissolved in DMSO and so this was added at an equivalent concentration (0.1 %) to wells containing untreated control cells. Following treatment, total RNA was extracted from the cells, cDNA synthesized and qPCR performed as described above using the following primers: *RALDH2* (NM_003888.3, NM_170696.2, NM_170697.2, NM_001206897.1): For, CAC TGA GCA GGG TCC CCA GAT TGA, Rev, AAC CCC TTT CGG CCC AGT CCT; *RALDH3* (NM_000693.3, NM_001293815.1): For, CGC AAC CTG GAG GTC AAG TTC ACC, Rev, AGC CTT GTC CAC GTC GGG CTT A. *GAPDH* was used as a reference gene.

## Results

### Cyp26 transcript expression in the adult rodent brain

**Fig. 1:**
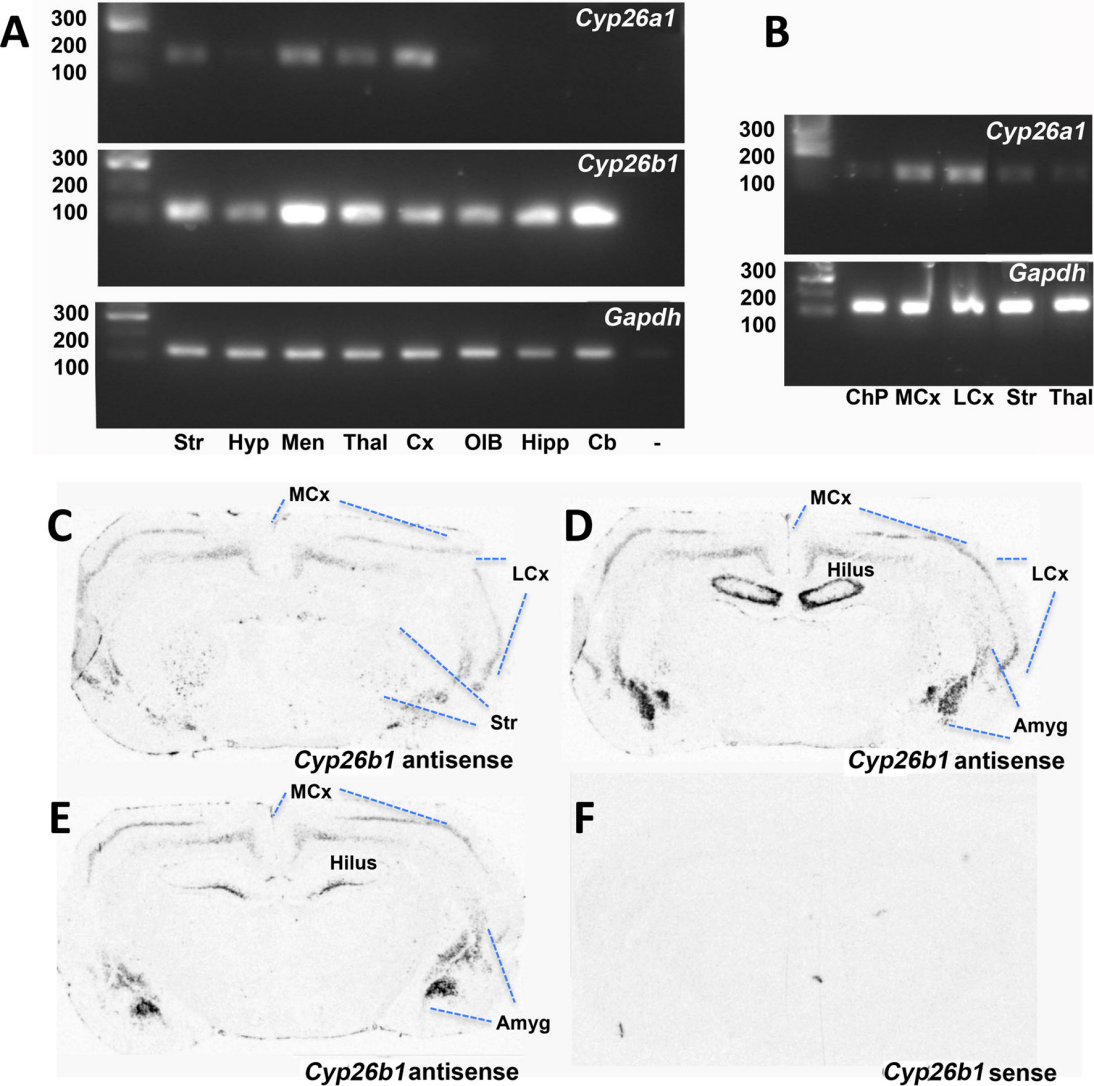
*Cyp26a1* and C*yp26b1* transcript expression in the adult rat brain. **a** RT-PCR of dissected rat tissue showed relatively weak bands for *Cyp26a1* in meninges, striatum, thalamus and cerebral cortex. *Cyp26b1* in contrast was stronger and more widespread with expression in striatum, olfactory bulb, hippocampus, hypothalamus, thalamus and cerebral cortex. **b** Expression of *Cyp26a1* in the rat brain was unexpected given that it is reported to be absent from the mouse brain. Rat tissues were thus dissected to avoid any contaminating meninges or choroid plexus as potential extraneous sources of the enzyme but *Cyp26a1* transcript was still detected in medial and lateral cortex and weakly present in the striatum. **c** *Cyp26b1*, the predominant Cyp26 in the rat brain, was examined by in situ hybridization and was present in localized regions of the cortex, amygdala, striatum and hippocampus. *Cb* cerebellum, *ChP* choroid plexus, *Cx* cortex, *Hyp* hypothalamus, *Hipp* hippocampus, *LCx* lateral cortex, *MCx* medial cortex, *Men* meninges, *OB* olfactory bulb, *Str* striatum, *Thal* thalamus

*Cyp26a1* gene expression is reported to be weak in the adult mouse brain (Abu-Abed et al. 2002) and PCR analysis of the adult rat brain (Fig. 1a) shows similar weak expression, with almost no *Cyp26a1* transcript detected in the hippocampus, hypothalamus, cerebellum and olfactory bulb. Some expression was detected in the meninges, striatum, thalamus and cerebral cortex (Fig. 1a). Dissection of tissue to avoid any contaminating meninges or choroid plexus found the levels in striatum and thalamus were low, and the choroid plexus itself had little expression, but the moderate amounts of *Cyp26a1* in the cortex were the same whether lateral or medial regions were dissected from caudal cortex (Fig. 1b). *Cyp26b1*, in contrast, is more strongly expressed and widely distributed in the rat brain than *Cyp26a1* and is particularly high in the cerebellum and meninges while also present in the striatum, olfactory bulb, hippocampus, hypothalamus, thalamus and cerebral cortex (Fig. 1a), extending an earlier description of *Cyp26b1* in the hippocampus and amygdala (Abu-Abed et al. 2002). *In situ* hybridization showed that, although *Cyp26b1* is widely distributed in the rat forebrain, it has a patterned rather than homogeneous distribution and is strongly expressed in subregions of the cortex, amygdala, striatum and hippocampus (Fig. 1c–e). The distribution of *Cyp26b1* suggests that this catabolic enzyme may play a role to pattern the distribution of RA in the adult brain, similar to its function in the developing embryo.

### A role for Cyp26b1 in patterning a paracrine action of retinoic acid in the rodent hippocampus

**Fig. 2:**
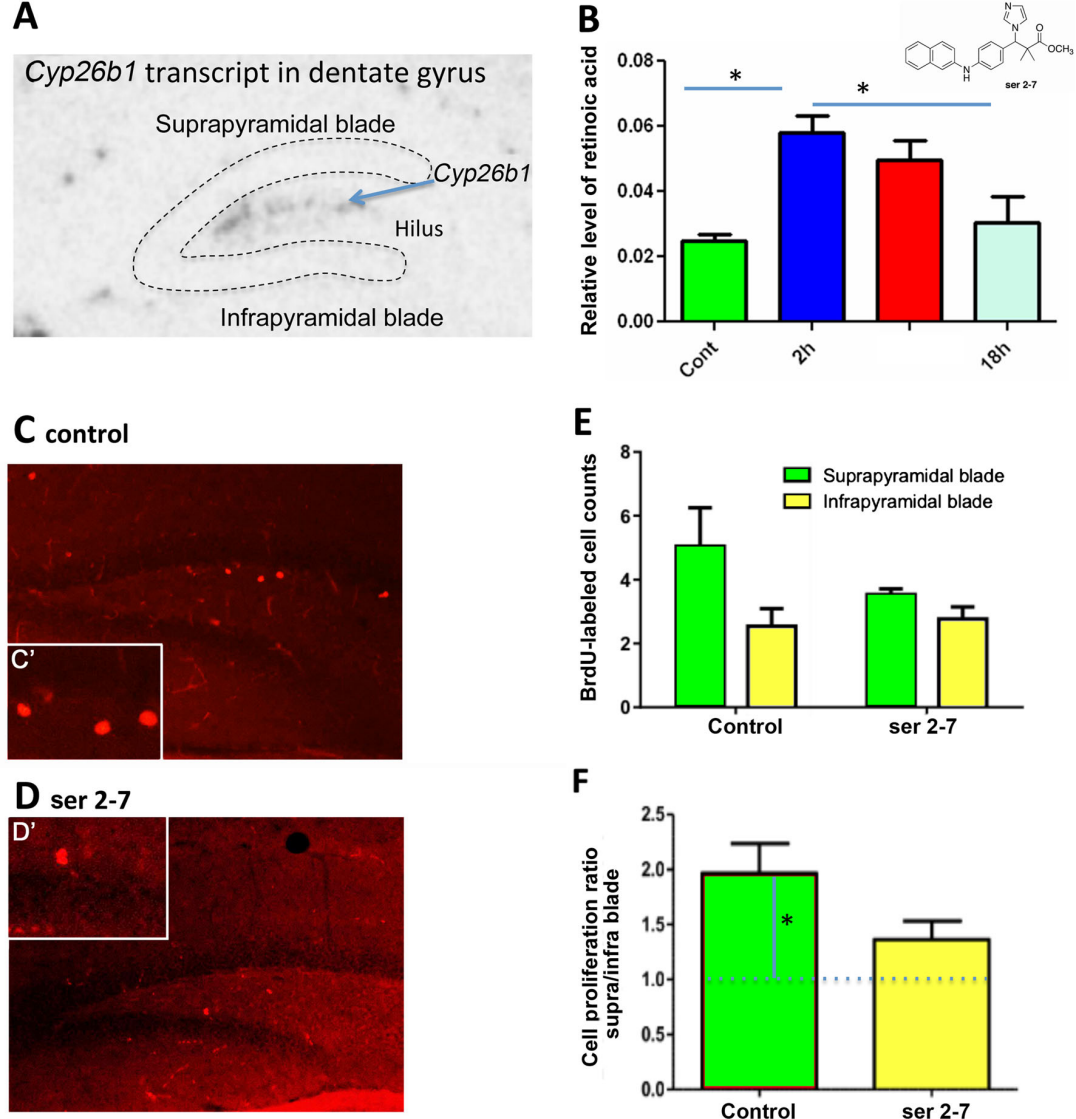
*Cyp26b1* is expressed in the hilus of the dentate gyrus and inhibition of Cyp26 alters the pattern of cell proliferation in the dentate gyrus. **a** In situ hybridization of C*yp26b1* illustrates expression of the catabolic enzymes in the hilus between the infrapyramidal (*lower*) and suprapyramidal (*upper*) blades of the dentate gyrus. **b** Relative levels of RA in the hippocampus 2, 6 and 18 h after injection of ser 2–7 (structure shown *top right*). RA levels were significantly increased 2 h post-injection, returning to pre-treatment levels by 18 h. * *P* < 0.05, mean ± SEM, *n* = 3. Cell proliferation in the subgranular zone (SGZ) of the dentate gyrus was then quantified after 3 days of treatment with ser 2–7. **C**, **d** Change in cell proliferation was determined in the SGZ by counting of BrdU-labeled cells with higher magnification views of individual cells shown in **c**’ and **d**’. **e** Quantification of BrdU-labeled cells in the SGZ showed that the number of BrdU-labeled cells in the suprapyramidal blade fell with ser 2–7 treatment, although not significantly. **f** When the ratio of BrdU-positive cells in the suprapyramidal versus the infrapyramidal blades of the dentate gyrus was determined it was significantly different from one in the control, vehicle-treated hippocampus (greater labeling in the suprapyramidal blade) but was not significantly different from one after ser 2–7 treatment (similar labeling in infrapyramidal and suprapyramidal blades)

In the dentate gyrus of the hippocampus (Fig. 2a), we have previously proposed that RA diffuses ventrally from the meninges to create a gradient of high RA in the infrapyramidal (lower) blade compared to suprapyramidal (upper) blade, which leads to a disparity in the rate of cell proliferation controlled by RA between the blades (Goodman et al. 2012). The location of Cyp26b1 between these blades will reduce diffusion of RA (Fig. 2a) and increase the steepness of the gradient. If this is the case then Cyp26b1 inhibition should reduce the difference in cell proliferation between the two blades. To test this hypothesis, we investigated the effect of Cyp26 inhibition on the rate of cell proliferation in the two blades of the dentate gyrus in the mouse (Goodman et al. 2012), treating mice with a new Cyp26 inhibitor, ser 2–7 (Fig. 2b), which has nearly 200-fold greater affinity for Cyp26 than previous inhibitors such as liarozole (Gomaa et al. 2011). Animals were sacrificed 2, 6 or 18 h after receiving a single IP injection of 10 mg/kg ser 2–7 or vehicle and the RA levels in the hippocampus analyzed using a cell-based bioassay. Treatment with ser 2–7 significantly increased hippocampal RA levels after 2–6 h (*P* < 0.05, ANOVA followed by post hoc Tukey’s HSD test). By 18 h post-injection, RA levels in the hippocampus of ser 2–7-treated mice had almost returned to control levels (Fig. 2b).

To examine the influence of this Cyp26 inhibitor on cell proliferation in the upper and lower blades of the dentate gyrus, mice were treated with ser 2–7 (10 mg/kg) or vehicle once a day for 3 days with the final dose injected 6 h before killing. Beginning 6 h before killing, animals also received 3 injections of the thymidine analog bromodeoxyuridine (BrdU) 2 h apart.

It was hypothesized that inhibition of Cyp26 by ser 2–7 would reduce the gradient of RA between the two blades and equalize cell proliferation between them. Proliferating cells were identified by incorporation of BrdU and cell proliferation was quantified by counting BrdU-positive nuclei (Fig. 2c, d). Ser 2–7 treatment decreased the number of BrdU-labeled cells per dentate gyrus, although this decline was not significant by ANOVA (*P* = 0.08, Fig. 2e). However, when analyzed as the ratio difference, significantly more BrdU-positive cells were observed in the upper blade of the dentate gyrus of vehicle-treated control animals than in the lower blade (Fig. 2f, ratio of suprapyramidal BrdU-positive cells/infrapyramidal BrdU-positive cells = 1.96; significantly different from a ratio of 1 by one-sample *t* test, *P* < 0.02, *n* = 5). Inhibition of Cyp26 function using ser 2–7 resulted in a ratio between the two blades closer to one (Fig. 2e, suprapyramidal/infrapyramidal BrdU ratio = 1.36; not significantly higher than a ratio of 1 by one-sample *t* test). The effect of ser 2–7 on cell proliferation is likely to be due to inhibition of Cyp26b1 activity in the region between the blades, resulting in more RA reaching the suprapyramidal blade. This may be combined with a systemic increase in RA due to whole-body/systemic inhibition of Cyp26, creating greater homogeneity of RA across the hippocampus. Overall this leads to suppression of RA catabolism in both blades and a ratio of cell proliferation between blades closer to one. These results point to a paracrine action of RA in the blades of dentate gyrus, where it acts at a distance from its site of synthesis in the meninges, as we have previously proposed (Goodman et al. 2012).

### CYP26 expression in the human brain: is paracrine signaling the only signaling route for RA in the brain?

**Fig. 3:**
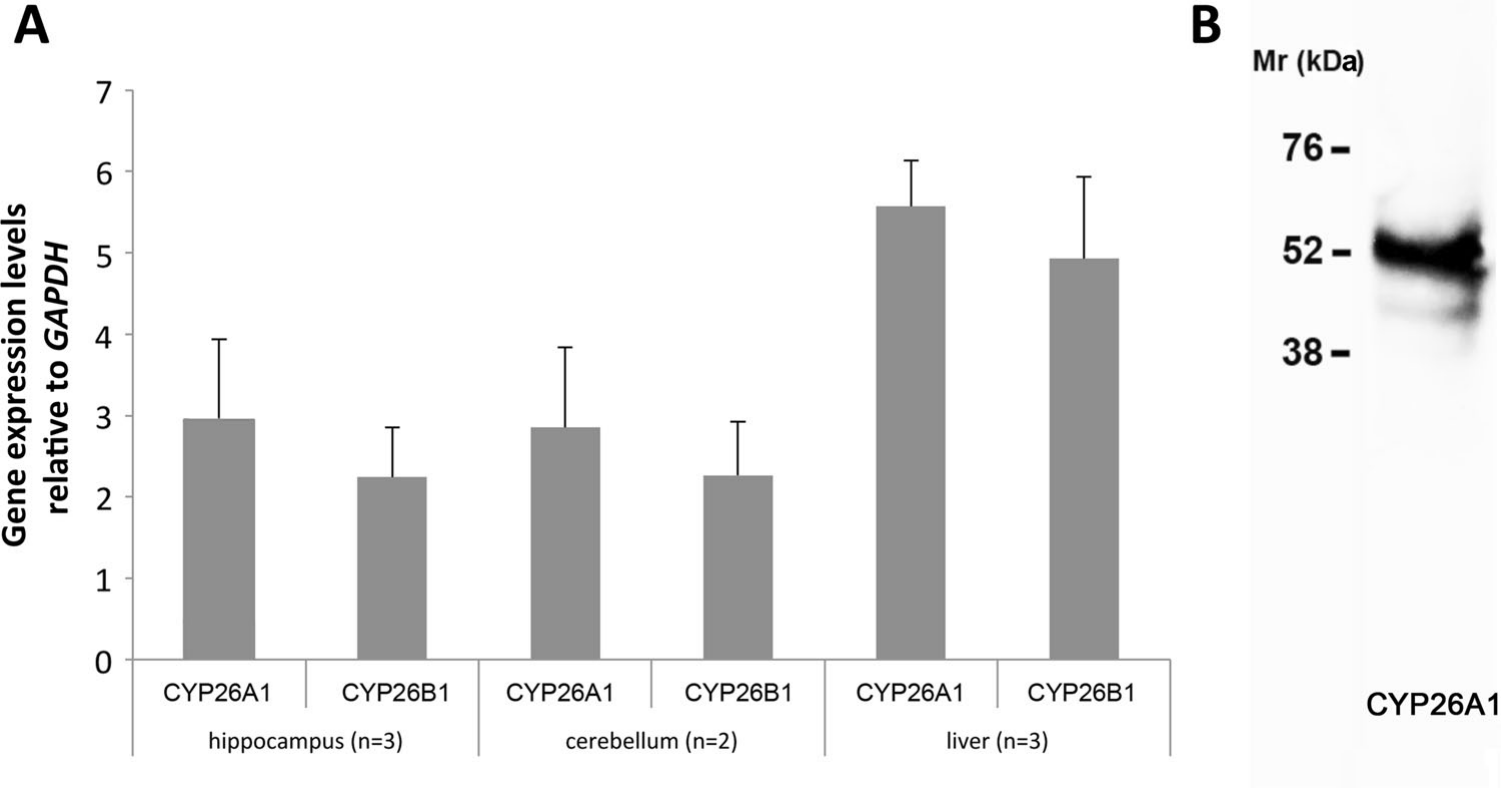
Expression of *CYP26A1* and *CYP26B1* in the human brain. **a** qPCR analysis of *CYP26A1* and *CYP26B1* showed expression in both the human hippocampus and cerebellum at levels approximately half of that in the human liver. **b** Western blotting for CYP26A1 protein in the human hippocampus identifies a prominent band of approximately 54 kDa

**Fig. 4:**
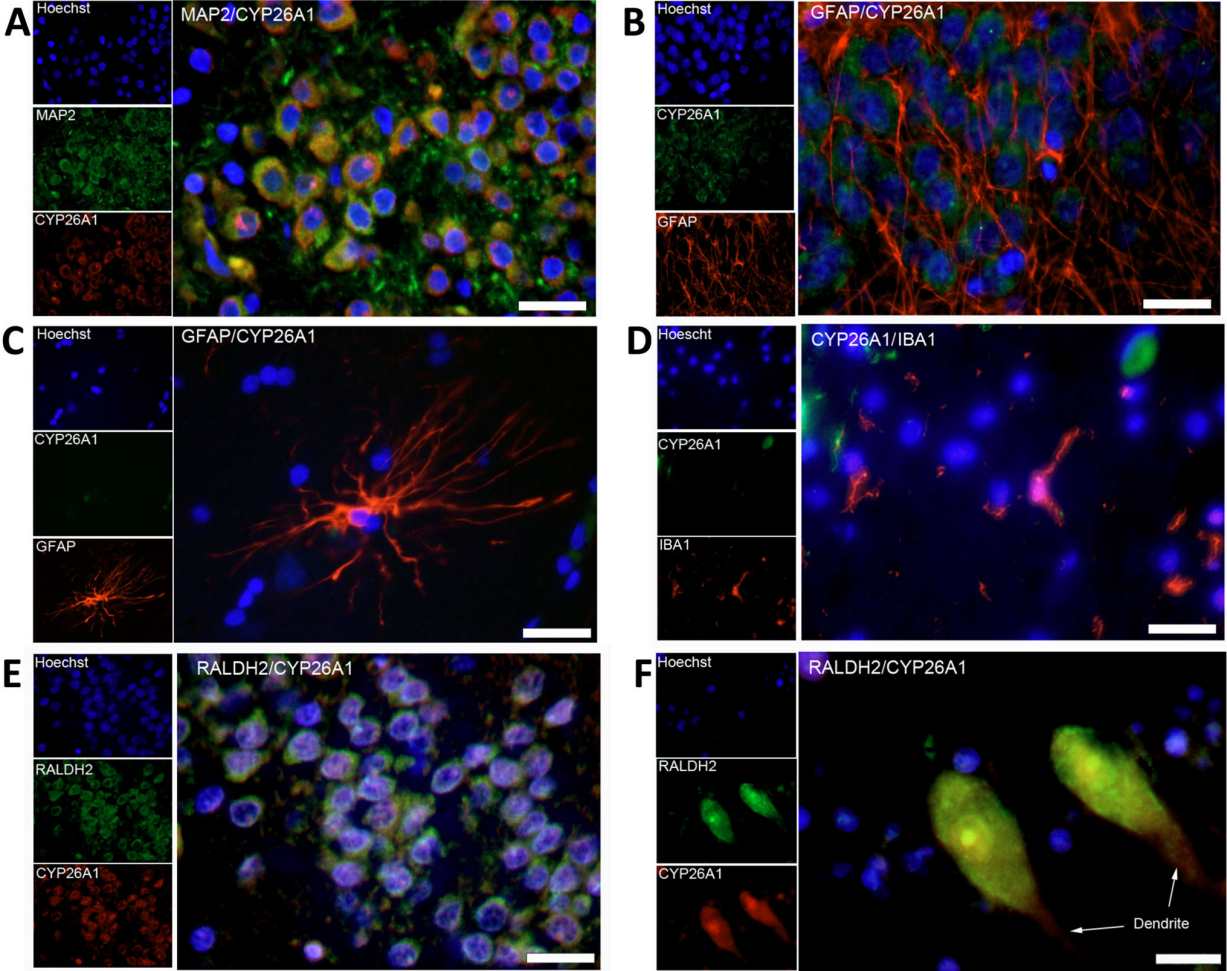
Immunohistochemistry of CYP26A1 in the human hippocampus and cerebellum. **a** Double-labeling of cells with antibodies to the neuron-specific protein MAP2 and CYP26A1 showed expression of this catabolic enzyme in neurons of the dentate gyrus. In contrast, double-labeling of cells with antibodies to GFAP showed that **b** glia in the dentate gyrus did not express CYP26A1 and **c** cells with astrocyte morphology in the CA1 hippocampal subfield also lacked CYP26A1. **d** Similarly, cells labeled with an antibody against the microglia-specific IBA1 did not express CYP26A1. **e**, **f** Double-labeling of cells for both CYP26A1 and RALDH2 showed that cells in the dentate gyrus (**e**) and cerebellum (**f**, Purkinje cells) co-expressed these enzymes

The greater size of the human compared to the rodent brain and the larger fiber tracts and hence the larger amount of lipid in the form of myelin may mean that establishing robust gradients of RA across, for instance, the dentate gyrus by diffusion from an external source is much harder. qPCR indicated that both *CYP26A1* and *CYP26B1* were present in the human hippocampus and cerebellum at comparable levels to that in liver (Fig. 3a). An antibody against CYP26A1 was identified that gave a single major band by western blot (Fig. 3b) of similar molecular weight to the 56 kDa previously reported (Topletz et al. 2012). Using this antibody for immunohistochemistry, it was found that, unlike the rat and mouse, MAP2-positive neurons in the human dentate gyrus express CYP26A1 (Fig. 4a, 95.0 % of MAP2-positive cells expressing CYP26A1). In contrast, GFAP positive astrocytes in the dentate gyrus (Fig. 4b) were negative for CYP26A1, as were the same cell type in CA1 of the hippocampus seen at higher magnification (Fig. 4c). Iba1-positive microglia, which in culture express the catabolic enzyme (Hellmann-Regen et al. 2013), were also negative for CYP26A1 (Fig. 4d). Double-labeling of human brain sections with antibodies against CYP26A1 and one of the RA synthetic enzymes, RALDH2, identified a further difference from the rodent brain as all cells in both the dentate gyrus and cerebellum co-expressed CYP26A1 and RALDH2 (Fig. 4e, f). Widespread co-expression of the synthetic and catabolic enzymes in hippocampal neurons, which are also known to express RA receptors (Fragoso et al. 2012), suggests that RA has an autocrine function in these cells rather than diffusing to neurons from an external source, as occurs in the rodent dentate gyrus (Goodman et al. 2012).

### Retinoid-regulated synthetic and catabolic enzyme expression in a human neuroblastoma cell line

**Fig. 5:**
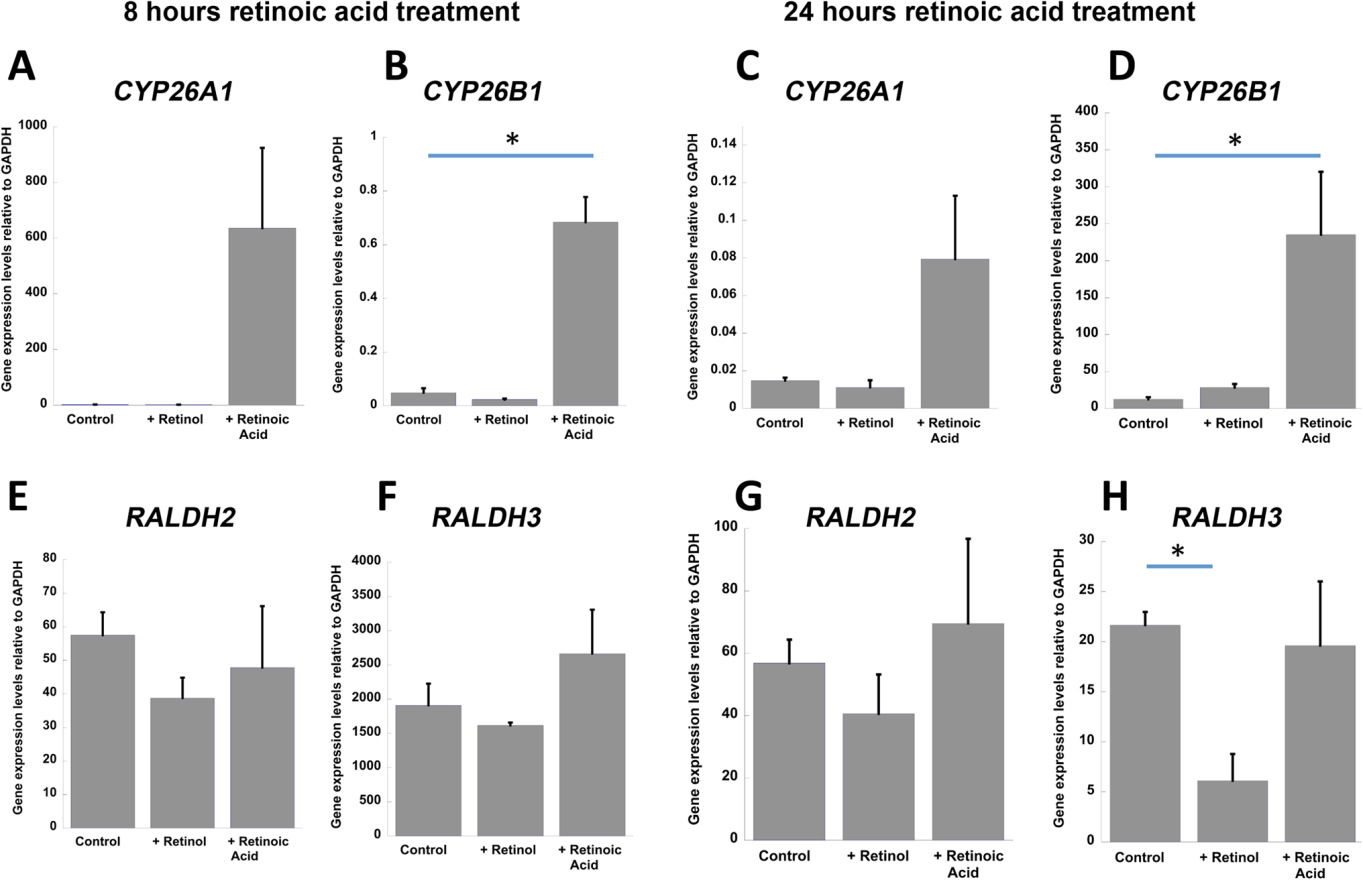
Regulation by RA and retinol of *CYP26* catabolic and *RALDH* synthetic enzymes transcript in SH-SY5Y cells. **a**, **b** Eight hours of RA treatment induced both *CYP26A1* (**a**) and *CYP26B1* expression (**b**, *P* = 0.042, ANOVA with Tukey’s post hoc test) whereas retinol had no effect. **c**, **d** Twenty-four hours of RA treatment similarly induced both *CYP26A1* (**c**) and *CYP26B1* (**d**, *P* = 0.0005, ANOVA with Tukey’s post hoc test) while retinol had no effect. **e**, **f** Eight hours of retinol or RA treatment did not result in significant change in *RALDH2* and *RALDH3*. **g**, **h** In contrast, *RALDH3* expression decreased significantly 24 h after retinol treatment (**h**, *P* = 0.033, ANOVA with Tukey’s post hoc test)

If human neurons can modulate intracellular RA levels by expressing both RA synthetic and catabolic enzymes then it would be expected that expression of the genes encoding the catabolic and synthetic enzymes may be altered in response to changes in vitamin A/RA levels, in order to rebalance retinoid levels. To explore this, SH-SY5Y human neuroblastoma cells were treated with RA or retinol (+ retinyl palmitate) for 8 or 24 h. Both *CYP26A1* (Fig. 5a, an over 600-fold increase but not significant because of a high variability in response) and *CYP26B1* (Fig. 5b, *P* = 0.042, ANOVA with post hoc Tukey’s test) were rapidly induced by RA, as has been reported previously (Ray et al. 1997), providing a mechanism by which excess RA can be removed. Both were also induced after 24 h of RA treatment, although only *CYP26B1* significantly (Fig. 5d, *P* = 0.0005, ANOVA with post hoc Tukey’s test). Addition of retinol to the medium had no effect on these catabolic enzymes (Fig. 5a–d). The RA synthetic enzymes *RALDH2* and *RALDH3* were also examined (an additional family member, *RALDH1*, was absent from SH-SY5Y cells, results not shown). In contrast to the catabolic enzymes, *RALDH2* and *RALDH3* did not respond to RA (Fig. 5e–h). However, the presence of retinol after 24 h triggered a significant repression of *RALDH3* (Fig. 5h, *P* = 0.033, ANOVA with post hoc Tukey’s test), potentially reducing RA synthesis when vitamin A levels are high but enabling these neurons to make more efficient use of reduced amounts when vitamin A levels are low.

## Discussion

Cyp26a1 and Cyp26b1 are the primary enzymes for RA catabolism in the body (McCaffery and Simons 2007; Ross and Zolfaghari 2011). Much initial attention was directed to their role in the developing embryo (Abu-Abed et al. 2001; Yashiro et al. 2004) where a key element to their function arose from their spatial distribution—expression in regions adjacent to areas of high RA synthesis, limiting diffusion to create precise patterns of RA concentration. The distribution of retinoic acid synthetic and catabolic enzymes in the embryo was always found to be complementary, with RA diffusing from synthetic tissue and acting as a paracrine factor on tissues distant from this source (Swindell et al. 1999). Initial findings in the adult rodent brain pointed to a similar paracrine action for RA.

In the rodent brain the predominant source of RA is the meninges surrounding the brain (Goodman et al. 2012). In situ hybridization shows the expression of the catabolic enzyme *Cyp26b1* in subregions of the brain. Expression is strong in several areas of the forebrain, presumably limiting the concentration of RA in these regions. Such regions of high *Cyp26b1* expression include the lateral and medial cortex, but it is absent from the upper layers of the medial cortex where the synthetic enzyme Raldh3 is present, potentially setting up regions of RA-regulated plasticity in the cortex (Wagner et al. 2006). There are limited regions of RA signaling in the amygdala where it is also proposed to delineate zones of plasticity (Thompson Haskell et al. 2002) and these regions would be assumed to be complementary to the intense areas of *Cyp26b1* expression in subregions of the amygdala. These patterns of *Cyp26* expression, separated from regions of RA synthesis, suggest a paracrine action of RA.

This paracrine function for RA is exemplified by its role in control of cell proliferation in neurogenic regions in the rodent hippocampus. *In situ* hybridization shows strong *Cyp26b1* expression in cells of the hilus between the two blades of the dentate gyrus in the hippocampus. We previously proposed that this reduces the amount of RA that can diffuse from the meninges adjacent to the infrapyramidal blade of the dentate gyrus to reach the upper, suprapyramidal blade. We have previously shown that this asymmetry in RA between the blades of the dentate gyrus contributes to the difference in rates of cell proliferation in the subgranular zones of the two blades. Neurogenesis in these regions is crucial for several aspects of hippocampus-dependent memory, including pattern separation (Schmidt et al. 2012), the process that reduces confusion of old memories with new. Differences in the functions of the two blades are also proposed to be important in the pattern separation task of the dentate gyrus (Schmidt et al. 2012). Normally the upper, suprapyramidal blade has significantly higher levels of cell proliferation than the lower blade with a two-fold ratio between the blades. Treatment of mice with the Cyp26 inhibitor ser 2–7 resulted in equalization of the ratio of BrdU incorporation between the upper and lower blades. In the normal animal, this may have a detrimental effect on pattern separation aspects of memory.

Although there may be a detrimental effect under normal circumstances, the effectiveness of ser 2–7 to increase RA in the hippocampus may have some benefit in disorders where RA signaling is believed to decline, such as Alzheimer’s disease. Several recent articles and reviews have proposed RA treatment for this disorder because of its action to promote the non-amyloidogenic pathway of amyloid precursor protein cleavage as well as its anti-inflammatory properties, promotion of cholinergic transmission, reduction of intracellular cholesterol and cell-protective properties (Ono and Yamada 2012; Sodhi and Singh 2014). Increasing hippocampal RA may be particularly beneficial because RA levels are declining in the aging hippocampus (Etchamendy et al. 2001). However, rather than administration of RA itself, the use of Cyp26 inhibitors to raise endogenous RA is a route less likely to result in excessively high and potentially damaging amounts of RA in the brain. Further, this method avoids the problem of retinoid resistance where addition of RA induces the Cyp26 enzymes (Ray et al. 1997), limiting the efficacy of RA-based treatments over time.

The study of CYP26 in the human, versus rodent, brain has revealed a wider distribution of the catabolic enzymes in the human, suggesting a more widespread action. We have previously shown that RA signaling in the human brain differed from that of the rodent with the RA synthetic enzymes RALDH1, 2 and 3 expressed not just in the surrounding meninges but also in many neurons (Fragoso et al. 2012), suggesting a higher requirement for RA in the human brain compared to the rodent. In this study qPCR confirmed expression of both *CYP26A1* and *CYP26B1* in the human brain. The methods employed did not provide an absolute comparison of the relative amounts but previous quantitative studies (Topletz et al. 2012) found about fivefold higher levels of *CYP26B1* than *CYP26A1* in the cerebral cortex, but in the cerebellum *CYP26B1* predominated by over 100-fold.

Given the expression of *CYP26A1* and *CYP26B1* transcripts in the human brain we investigated the distribution of CYP26A1 protein, for which we selected an antibody which gave only a single band by western blotting. Immunohistochemistry indicated that, like RALDH2 (Fragoso et al. 2012), CYP26A1 was present in neurons. Comparison of CYP26A1 and RALDH2 expression indicated a high degree of colocalization in granule neurons of the hippocampus and Purkinje neurons of the cerebellum. We have previously demonstrated that these neurons express RA receptors and their co-expression of both RA synthetic and catabolic enzymes implies the capacity for autocrine RA signaling with the ability to both upregulate and downregulate the RA signal. If this were the case it would be expected that the synthetic and catabolic enzymes would respond to relative levels of RA and its retinol precursor. Expression of the genes encoding the RALDH and CYP26 enzymes was examined in the SH-SY5Y neuroblastoma cell line. As previously reported in many studies on non-neural cells [for example (Ray et al. 1997; Zhang et al. 2010)], RA rapidly induces *CYP26A1* and *CYP26B1*, although this induction is attenuated by 24 h. In contrast, transcript levels of the synthetic enzymes *RALDH2* and *RALDH3* were not influenced by RA; however, when retinol levels were increased there was a significant fall in *RALDH3* transcript after 24 h. Such feedback control by the substrate for RA has not been previously described for the synthetic enzymes and suggests a mechanism that could help maintain local RA levels in the brain relatively constant by helping to prevent wide fluctuations.

## Summary

In the rodent brain RA can, as a paracrine signaling factor, diffuse across tissues to control localized gene expression as it does in the embryo. The Cyp26 catabolic enzymes, expressed in cells distant from the site of synthesis, help to regulate the pattern of RA diffusion. This separation between sites of synthesis and catabolism occurs in other adult rodent tissues such as the uterus (Ma et al. 2012). However, in human neurons RA can have a different, autocrine, function that is restrained by CYP26. Neurons, but not glia, in the adult human hippocampus and cerebellum express both CYP26A1 and RALDH2 which together can modulate the balance of RA within single cells. This contrasts with the embryo and adult rodent brain in which the synthetic and catabolic enzymes control the balance of RA broadly across tissues. This would be the first time that an autocrine RA signaling system, with the capacity to switch on and off the RA signal in individual neurons, has been proposed in the human brain.

## References

[CR1] Abu-Abed S, Dolle P, Metzger D, Beckett B, Chambon P, Petkovich M (2001). The retinoic acid-metabolizing enzyme, CYP26A1, is essential for normal hindbrain patterning, vertebral identity, and development of posterior structures. Genes Dev.

[CR2] Abu-Abed S, MacLean G, Fraulob V, Chambon P, Petkovich M, Dolle P (2002). Differential expression of the retinoic acid-metabolizing enzymes CYP26A1 and CYP26B1 during murine organogenesis. Mech Dev.

[CR3] Aoto J, Nam CI, Poon MM, Ting P, Chen L (2008). Synaptic signaling by all-trans retinoic acid in homeostatic synaptic plasticity. Neuron.

[CR4] Bowles J, Knight D, Smith C, Wilhelm D, Richman J, Mamiya S, Yashiro K, Chawengsaksophak K, Wilson MJ, Rossant J, Hamada H, Koopman P (2006). Retinoid signaling determines germ cell fate in mice. Science.

[CR5] Chen N, Napoli JL (2008). All-trans-retinoic acid stimulates translation and induces spine formation in hippocampal neurons through a membrane-associated RARalpha. FASEB J.

[CR6] Chithalen JV, Luu L, Petkovich M, Jones G (2002). HPLC-MS/MS analysis of the products generated from all-trans-retinoic acid using recombinant human CYP26A. J Lipid Res.

[CR7] Etchamendy N, Enderlin V, Marighetto A, Vouimba RM, Pallet V, Jaffard R, Higueret P (2001). Alleviation of a selective age-related relational memory deficit in mice by pharmacologically induced normalization of brain retinoid signaling. J Neurosci.

[CR8] Fragoso YD, Shearer KD, Sementilli A, de Carvalho LV, McCaffery PJ (2012). High expression of retinoic acid receptors and synthetic enzymes in the human hippocampus. Brain Struct Funct.

[CR9] Gomaa MS, Bridgens CE, Aboraia AS, Veal GJ, Redfern CP, Brancale A, Armstrong JL, Simons C (2011). Small molecule inhibitors of retinoic acid 4-hydroxylase (CYP26): synthesis and biological evaluation of imidazole methyl 3-(4-(aryl-2-ylamino)phenyl)propanoates. J Med Chem.

[CR10] Goodman T, Crandall JE, Nanescu SE, Quadro L, Shearer K, Ross A, McCaffery P (2012). Patterning of retinoic acid signaling and cell proliferation in the hippocampus. Hippocampus.

[CR11] Helfer G, Ross AW, Russell L, Thomson LM, Shearer KD, Goodman TH, McCaffery PJ, Morgan PJ (2012). Photoperiod regulates vitamin A and Wnt/beta-catenin signaling in F344 rats. Endocrinology.

[CR12] Hellmann-Regen J, Kronenberg G, Uhlemann R, Freyer D, Endres M, Gertz K (2013). Accelerated degradation of retinoic acid by activated microglia. J Neuroimmunol.

[CR13] Kuhn HG, Winkler J, Kempermann G, Thal LJ, Gage FH (1997). Epidermal growth factor and fibroblast growth factor-2 have different effects on neural progenitors in the adult rat brain. J Neurosci.

[CR14] Ma JJ, Han BC, Yang Y, Peng JP (2012). Retinoic acid synthesis and metabolism are concurrent in the mouse uterus during peri-implantation. Cell Tissue Res.

[CR15] Makitie LT, Kanerva K, Polvikoski T, Paetau A, Andersson LC (2013). Brain neurons express ornithine decarboxylase-activating antizyme inhibitor 2 with accumulation in Alzheimer’s disease. Brain Pathol.

[CR16] McCaffery P, Simons C (2007). Prospective teratology of retinoic acid metabolic blocking agents (RAMBAs) and loss of CYP26 activity. Curr Pharm Des.

[CR17] Napoli JL (2012). Physiological insights into all-trans-retinoic acid biosynthesis. Biochim Biophys Acta.

[CR18] Neumann M, Gabel D (2002). Simple method for reduction of autofluorescence in fluorescence microscopy. J Histochem Cytochem.

[CR19] Ono K, Yamada M (2012). Vitamin A and Alzheimer’s disease. Geriatri Gerontol Int.

[CR20] Ray W, Bain G, Yao M, Gottlieb DI (1997). CYP26, a novel mammalian cytochrome P450, is induced by retinoic acid and defines a new family. J Biol Chem.

[CR21] Ross AC, Zolfaghari R (2011). Cytochrome P450 s in the regulation of cellular retinoic acid metabolism. Annu Rev Nutr.

[CR22] Ross AW, Johnson CE, Bell LM, Reilly L, Duncan JS, Barrett P, Heideman PD, Morgan PJ (2009). Divergent regulation of hypothalamic neuropeptide Y and agouti-related protein by photoperiod in F344 rats with differential food intake and growth. J Neuroendocrinol.

[CR23] Rossant J, Zirngibl R, Cado D, Shago M, Giguère V (1991). Expression of a retinoic acid response element-hsplacZ transgene defines specific domains of transcriptional activity during mouse embryogenesis. Genes Dev.

[CR24] Schmidt B, Marrone DF, Markus EJ (2012). Disambiguating the similar: the dentate gyrus and pattern separation. Behav Brain Res.

[CR25] Schnell SA, Staines WA, Wessendorf MW (1999). Reduction of lipofuscin-like autofluorescence in fluorescently labeled tissue. J Histochem Cytochem.

[CR26] Slavotinek AM, Mehrotra P, Nazarenko I, Tang PL, Lao R, Cameron D, Li B, Chu C, Chou C, Marqueling AL, Yahyavi M, Cordoro K, Frieden I, Glaser T, Prescott T, Morren MA, Devriendt K, Kwok PY, Petkovich M, Desnick RJ (2013). Focal facial dermal dysplasia, type IV, is caused by mutations in CYP26C1. Hum Mol Genet.

[CR27] Sodhi RK, Singh N (2014). Retinoids as potential targets for Alzheimer’s disease. Pharmacol Biochem Behav.

[CR28] Swindell EC, Thaller C, Sockanathan S, Petkovich M, Jessell TM, Eichele G (1999). Complementary domains of retinoic acid production and degradation in the early chick embryo. Dev Biol.

[CR29] Thompson Haskell G, Maynard TM, Shatzmiller RA, Lamantia AS (2002). Retinoic acid signaling at sites of plasticity in the mature central nervous system. J Comp Neurol.

[CR30] Topletz AR, Thatcher JE, Zelter A, Lutz JD, Tay S, Nelson WL, Isoherranen N (2012). Comparison of the function and expression of CYP26A1 and CYP26B1, the two retinoic acid hydroxylases. Biochem Pharmacol.

[CR31] Wagner M, Han B, Jessell TM (1992). Regional differences in retinoid release from embryonic neural tissue detected by an in vitro reporter assay. Development.

[CR32] Wagner E, Luo T, Sakai Y, Parada LF, Drager UC (2006). Retinoic acid delineates the topography of neuronal plasticity in postnatal cerebral cortex. Eur J Neurosci.

[CR33] Yashiro K, Zhao X, Uehara M, Yamashita K, Nishijima M, Nishino J, Saijoh Y, Sakai Y, Hamada H (2004). Regulation of retinoic acid distribution is required for proximodistal patterning and outgrowth of the developing mouse limb. Dev Cell.

[CR34] Ye J, Coulouris G, Zaretskaya I, Cutcutache I, Rozen S, Madden TL (2012). Primer-BLAST: a tool to design target-specific primers for polymerase chain reaction. BMC Bioinformatics.

[CR35] Zhang Y, Zolfaghari R, Ross AC (2010). Multiple retinoic acid response elements cooperate to enhance the inducibility of CYP26A1 gene expression in liver. Gene.

